# Spatiotopic and retinotopic memory in the context of natural images

**DOI:** 10.1167/jov.22.4.11

**Published:** 2022-03-24

**Authors:** Noah J. Steinberg, Zvi N. Roth, Elisha P. Merriam

**Affiliations:** 1 Laboratory of Brain and Cognition, National Institute of Mental Health, NIH, Bethesda, MD, USA

**Keywords:** spatiotopy, memory, saccades

## Abstract

Neural responses throughout the visual cortex encode stimulus location in a retinotopic (i.e., eye-centered) reference frame, and memory for stimulus position is most precise in retinal coordinates. Yet visual perception is spatiotopic: objects are perceived as stationary, even though eye movements cause frequent displacement of their location on the retina. Previous studies found that, after a single saccade, memory of retinotopic locations is more accurate than memory of spatiotopic locations. However, it is not known whether various aspects of natural viewing affect the retinotopic reference frame advantage. We found that the retinotopic advantage may in part depend on a retinal afterimage, which can be effectively nullified through backwards masking. Moreover, in the presence of natural scenes, spatiotopic memory is more accurate than retinotopic memory, but only when subjects are provided sufficient time to process the scene before the eye movement. Our results demonstrate that retinotopic memory is not always more accurate than spatiotopic memory and that the fidelity of memory traces in both reference frames are sensitive to the presence of contextual cues.

## Introduction

The visual system is primarily retinotopic. Throughout the brain, positions of visual stimuli are encoded according to their location on the retina (Gardner, Merriam, Movshon, & Heeger, 2008; Golomb & Kanwisher, 2012a). Eye movements pose a problem for a retinotopic system: with each eye movement, stationary objects in the world change position on the retina, and yet we do not perceive these displacements. How does the brain construct a stable percept from this unstable retinal input? One possibility is that the brain updates, or “remaps,” the coordinates of salient visual stimuli in conjunction with each eye movement (Duhamel, Colby, & Goldberg, 1992; Golomb & Mazer, 2021; Merriam & Colby, 2005). This idea has gained considerable support over the last decades from physiological recordings (Duhamel et al., 1992; Neupane, Guitton, & Pack, 2016a, 2016b), human functional imaging (Fairhall, Schwarzbach, Lingnau, Van Koningsbruggen, & Melcher, 2017; Merriam, Genovese, & Colby, 2003, 2007), and behavioral studies (Golomb & Mazer, 2021; Melcher, 2007).

A behavioral demonstration of remapping comes from studies in which observers were asked to remember the location of visual stimuli that were presented before an eye movement (Golomb & Kanwisher, 2012b; Shafer-Skelton & Golomb, 2018). One implication of the remapping hypothesis is that coordinate transformations occurring in conjunction with a saccade may be inherently noisy, resulting in a degradation in the fidelity of spatial memory from one eye movement to the next. Consistent with this idea, after an eye movement, observers were found to be more accurate when asked to report the location in which stimuli appeared on the retina (i.e., a location that was not remapped) than when asked to report the location of the stimuli on the screen (i.e., the spatiotopic location, which required remapping) (Golomb & Kanwisher, 2012b). This result implies that retinal coordinates are the native reference frame of the visual system, and additional computations are performed by the brain, such as remapping, that actively construct a spatiotopic coordinate system, which is stable across eye movements. This transformation is imperfect and subject to internal noise, and results in a less accurate spatiotopic memory trace. This logic also predicts that the accuracy of spatiotopic memory should degrade as observers make additional saccades, as the error associated with each subsequent transformation accumulates (Collins, 2010; Golomb & Kanwisher, 2012b).

The goal of our study was to test the generality of the retinotopic memory advantage. Most psychophysical studies of remapping are conducted in highly controlled conditions that differ from naturalistic viewing conditions in several important respects. For example, it is common for subjects to be seated in a dark room with no visible objects aside from a fixation point and a high-contrast memory cue, with even the dark edge of the computer screen blending with the darkened room. But in real-world viewing conditions, important objects typically appear in the context of a visual scene that includes multiple salient visual landmarks that are stable across repeated eye movements. These nontarget objects in natural scenes may help the visual system to triangulate the target location. A second consideration has to do with the persistence of retinal activity after the offset of a stimulus. A high-contrast memory cue appearing in a dark room often leads to a retinal afterimage that can be used to guide retinotopic spatial judgements, long after the cue has disappeared. Such afterimages may be less of a factor when the memory cue and the background have a more similar luminance, as is often the case in naturalistic viewing.

We carried out a series of location memory experiments. We hypothesized that changing the visual landmarks and manipulating the strength of the retinal afterimage would affect the degree to which retinotopic memory outperforms spatiotopic memory. We tested this hypothesis in a series of five experiments in which subjects performed a spatiotopic and retinotopic memory task. In each experiment, we manipulated a different variable that we hypothesized might contribute to the fidelity of retinotopic and spatiotopic memory fidelity. In Experiment 1, subjects performed the task against a gray background. In Experiment 2, subjects were presented with a mask that diminished the impact of a retinal afterimage. In Experiments 3 through 5, we tested the role of visual contextual information. We found that spatial memory depends critically on both visual context and the retinal afterimage. Although the advantage of retinotopic memory was replicated under typical conditions, attenuating the retinal afterimage or including visual landmarks eliminated the advantage and even reversed it. In Experiment 6, we investigated whether proximity of the memory cue to the screen edge could systematically bias responses, conferring an advantage for retinotopic memory. Our results suggest that the reliance of the visual system on remapping mechanisms may depend on visual context.

## Methods

**Figure 1.**
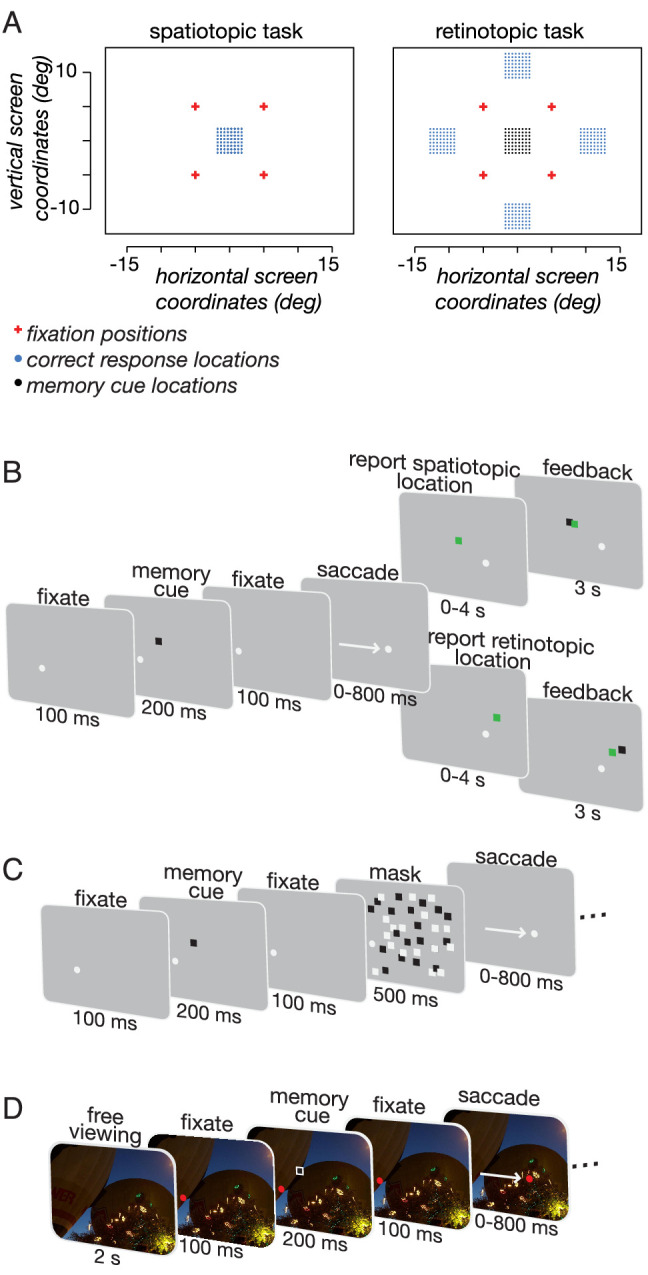
Experimental design. (A) Experiment design for the spatiotopic and retinotopic tasks. For the spatiotopic task, the memory cue locations (black dots) are at the same physical location as the correct memory locations (blue dots), so the black dots are hidden behind the blue in this panel. (B–D) Example trials for Experiments 1 through 3. Observers were instructed to fixate on the white dot until a black memory cue flashed on the screen. After another brief fixation period, observers made a saccade to the new location of the memory cue. A successful saccade within 800 ms prompted the mouse to appear on the screen, signaling observers to move the mouse to the remembered location. A green feedback square indicated the correct response location. (C) In Experiment 2, a salient visual mask appeared after the memory cue. (D) In Experiment 3, a naturalistic image background appeared before each trial. The fixation dot was red to make it easier for observers to find on the screen.

We measured the accuracy of retinotopic and spatiotopic memory after saccadic eye movements under a number of stimulus conditions (Experiment 1–6). The experimental protocol was adapted from Golomb and Kanwisher (2012b). Observers fixated a white dot (0.2°) for 100 to 300 ms. Subjects were required to fixate the dot within 200 ms, or else the trial was aborted. After successful fixation, a memory cue (0.8° × 0.8° black square) flashed on the screen for 200 ms. The memory cue location was randomly selected from a 3.5° square grid of potential locations at the center of the screen. An auditory tone was presented coincident with the memory cue, but was not task relevant. After a 500-ms delay, the fixation spot moved to another location on the screen, prompting a visually guided saccade. The fixation spot trajectory was determined by a pseudorandom sequence arranged around a 2 × 2 matrix of possible fixation points 11° apart and centered on the screen (Figure 1). Observers were required to make a saccade within 800 ms or else the trial was aborted. Once observers made a saccade to the new fixation spot location, the mouse pointer appeared at the saccade end point, and observers were instructed to use a computer mouse to click on the location where the memory cue had flashed. For the duration of the trial, including when making their response, observers were required to maintain fixation within 0.8 visual degrees.

Observers were instructed to indicate either the retinotopic or spatiotopic coordinate of the cue. In the retinotopic task, observers remembered the memory cue's location relative to their gaze position. In the spatiotopic task, observers were instructed to remember the memory cue's location relative to the screen. For each of the experiments, one-half of the subjects were randomly selected to complete the retinotopic task before completing the spatiotopic task, and half of them completed the experiment in the opposite order. The memorized location of the cue was indicated by a mouse click. Observers were given a maximum of 4 seconds to respond. Finally, the correct memory cue appeared on the screen, as well as a green feedback square (also 0.8° × 0.8°) to indicate their accuracy, which remained on the screen for 3 or 4 seconds before the beginning of the next trial. Observers’ eye position was monitored throughout the experiment. Every observer was required to complete a total of 200 (Experiment 1–3 and 6) or 270 (Experiment 4 and 5) correct trials within a single session.

### Experiment 1: No visual landmarks

Observers were instructed to remember the location of a memory cue while maintaining fixation on a small white dot (0.2°). In a single session, observers completed 5 blocks of 40 trials each. Each observer (*n* = 8) completed the retinotopic and spatiotopic tasks in separate sessions.

### Experiment 2: Masked cue

This experiment was designed to test the influence of the retinal afterimage of the memory cue on spatiotopic and retinotopic accuracy, following the protocol in experiment 3 of Golomb and Kanwisher (2012b). The design of this experiment was the same as Experiment 1, except for a salient visual mask that was presented for 500 ms after a 100-ms gap after the offset of the memory cue (Figure 1C). The mask consisted of 2,000 black and white squares (0.8°) that filled the screen. The fixation dot was displayed on top of the mask and observers (*n* = 8) were required to maintain fixation during presentation of the mask.

### Experiment 3: Natural scene background

The goal of this experiment was to test the influence of naturalistic images on retinotopic and spatiotopic memory. The design was similar to Experiment 1, with two critical differences. First, the screen was filled with a unique background image, rather than uniform gray. Second, the image appeared 2 seconds before the onset of the trial, during which observers were allowed to freely view the screen, and lasted until the beginning of the next trial. Once the 2-second free viewing time elapsed, the fixation dot appeared on the screen; observers were required to saccade there within 500 ms to begin the next phase of the trial. Observers (*n* = 9) completed 200 trials per session. Retinotopic and spatiotopic trials were conducted in separate sessions. Images for this experiment were taken from two separate databases: MIT300 (http://saliency.mit.edu/results_mit300.html) and MIT Database (http://people.csail.mit.edu/tjudd/WherePeopleLook/index.html). A subset of images from these two databases were selected if they had a horizontal aspect ratio and sufficient resolution to fill the screen. The fixation dot was red (instead of white, as in Experiment 1) so observers could more easily locate the fixation dot against the background images.

### Experiment 4: Variable background

The goal of Experiments 4 and 5 was to test whether the spatiotopic advantage observed with natural image backgrounds in Experiment 3 depends on the amount of time observers were able to view the image before the start of the trial. Observers (*n* = 8) completed this experiment (both the retinotopic and spatiotopic versions) with the ability to freely view the screen for 2 seconds before trial onset. We hypothesized that free viewing before trial onset would assist spatiotopic memory when there was a background image, but not when there was a gray screen with no spatial landmarks.

In each session of this experiment, one-third of the trials had a grey background (as in Experiment 1), one-third of the trials had an image background (as in Experiment 3), and one-third of the trials had a textured background created by phase-scrambling the natural images. Observers completed 270 trials per session, 90 trials per each of three conditions. The order of the three conditions was randomized across trials. A subset of images selected from the pool of images used in Experiment 3 were used in Experiment 4. Texture patterns were created by computing a Fourier transform of the images, permuting the phase, and then computing the inverse Fourier transform. This procedure preserved the image statistics while removing the semantically identifiable content from the images. In each session, observers performed either the retinotopic or spatiotopic memory task with backgrounds consisting of naturalistic images, their phase-scrambled counterparts, and uniform backgrounds. When observers made a fixation error on a trial, data from that trial were discarded. When an error occurred on either a naturalistic or a phase scrambled image, data from both versions of the image were discarded. The fixation dot in this experiment was colored red, just as in Experiment 3, for all trials regardless of the type of background. A white border (0.1° thickness) was added around the black memory cue to make it easier to see against the image backgrounds.

### Experiment 5: No viewing time

This experiment was identical to Experiment 4 (i.e., similar distribution and number of trials), with only one critical difference: in Experiment 5, there was no free viewing period before the start of each trial. Each trial immediately followed the previous one, requiring observers to maintain fixation without the time or ability to visually scan the background image.

### Experiment 6: Memory cue presented near edge of screen

This experiment was designed to test the influence of possible spatial biases in comparing retinotopic and spatiotopic memory. In all other experiments in this study, the memory cue was presented in the middle of the screen so that the correct retinotopic response was located near the screen edge, while the spatiotopic remained in the middle of the screen. However, if there were systematic biases favoring the retrieval of peripheral screen locations from memory or initiating motor responses to those locations, that would confer a retinotopic advantage. To test for this possibility, in Experiment 6, the memory cue was presented near the edge of the screen. The response in the spatiotopic task remained at the edge of the screen, whereas the response for the retinotopic task was always toward the center of the screen. The distributions of the distances between the memory cue and the fixation points were identical to all other experiments in this study. In all six experiments, observes performed 20 practice trials before the main experiment to ensure that they understood the task. Data from these practice trials were not analyzed.

### Subjects

A total of 36 observers with normal or corrected-to-normal vision participated in the study. Observers provided written informed consent in compliance with the Institutional Review Board at National Institutes of Health. Eight observers participated in Experiment 1 (6 female; mean age, 23.5 years; range, 23–25 years), eight observers in Experiment 2 (5 female; mean age, 23.8 years; range, 19–37 years), nine observers in Experiment 3 (6 female; mean age, 23.5 years; age range, 22–28 years), eight observers in Experiment 4 with no free viewing (4 female; mean age, 23.6 years; age range, 22–28 years), eight observers in Experiment 5 with free viewing (6 female; mean age, 24.5 years; age range, 22–30 years), and eight observers in Experiment 6 (7 female; mean age, 23.0 years; age range, 21–25 years). A total of nine observers participated in two different experiments and two observers participated in three experiments. In total, there were 49 sessions across the six experiments.

### Experimental setup

Stimuli were generated using Matlab (MathWorks, Natick, MA) and MGL (Gardner, Merriam, Schluppeck, & Larsson, 2018) on a Macintosh computer, and presented on a 61-inch screen (benq XL242OZ) positioned on a table 57 cm in front of the observer. Observers were seated in a darkened room with sound attenuation. An Eyelink 1000 eye-tracking system was used to measure binocular eye position at 1,000 Hz. Eyelink calibration was performed at the beginning of the session and repeated intermittently throughout the session to ensure that eye tracking accuracy remained within 1° of visual angle throughout the experiment.

### Analyses

Embedded within the stimulus presentation code were several criteria for correct task performance, including the following: 1) observers were required to make a saccade within 800 ms after the movement of the fixation spot; 2) observers were required to make a mouse response within 4 seconds after the instruction to do so; and 3) observers were required to maintain fixation within 0.8° at all times during the trial. If any of these conditions were not met, the trial was flagged as an error, a tone alerted the observer, and the trial was immediately discarded and replaced. Neither memory cue nor the feedback (if the observer clicked the mouse before fixation was lost) reappeared on the screen on discarded trials. Hence, all trials that were included in the analysis met fixation and saccade requirements. For each trial, memory error was calculated as the Euclidean distance between the reported answer and the correct location. Following Golomb and Kanwisher (2012b), we discarded trials with an error of more than 5.5°, because these responses were in the wrong region of the screen. Across all experiments, 95.3% of all trials met all inclusion criteria and were used for analysis.

## Results

### Experiment 1

**Figure 2.**
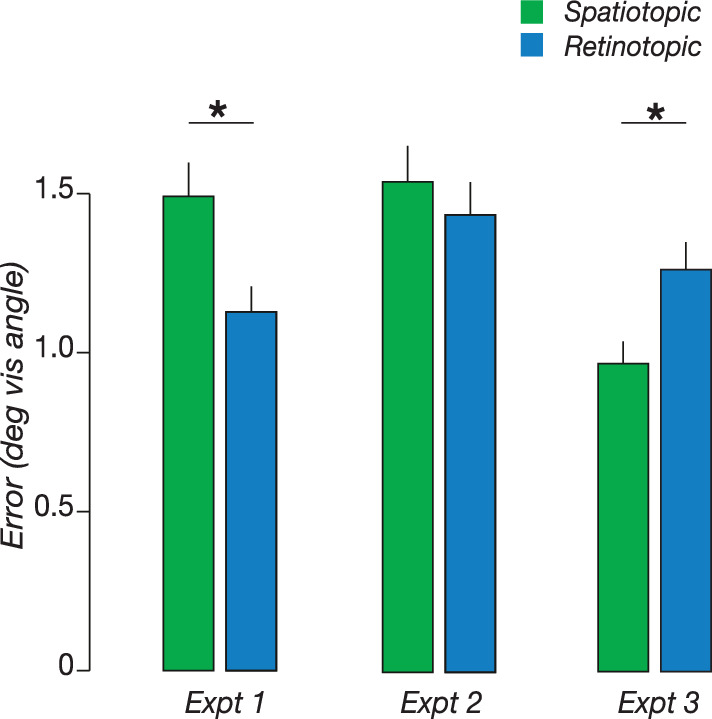
Memory accuracy in experiment 1 (gray background), experiment 2 (gray background with mask), and experiment 3 (image background). * indicate p < 0.05.

In this experiment, we tested the accuracy of spatial memory after an eye movement when observers were asked to remember either the retinotopic or the spatiotopic location of a memory cue. Following previous reports (Golomb & Kanwisher, 2012b; Shafer-Skelton & Golomb, 2018), we found that retinotopic memory was more accurate than spatiotopic memory (Cohen's d 1.616, t(7) = 4.66, *P* = 0.002; Figure 2, Table 1). Similarly, retinotopic precision (0.62 error) was higher than spatiotopic precision (0.78 error; t(7) = 2.99, *P* = 0.02). The accuracy and precision advantage of retinotopic memory in our experiment was commensurate with previous reports (Golomb & Kanwisher, 2012b).

**Table 1.** Statistics for each experiment.

Experiment number	Background	Free viewing time (seconds)	Sample size (*n*)	Presence of a mask?	Memory cue presented near screen edge	Degrees of freedom	Cohen's d (retinotopic accuracy larger than spatiotopic)	T value	*P* value
1	Gray screen	0	8			7	1.616	4.66	0.002
2	Gray screen	0	8	X		7	0.288	0.86	0.42
3	Natural images	2	9			8	−1.585	−3.85	0.002
4	Gray screen	2	8			7	0.346	0.98	0.36
4 (cont)	Natural images	2	8			7	−0.870	−2.46	0.04
4 (cont)	Scrambled	2	8			7	0.462	1.31	0.23
5	Gray screen	0	8			7	0.774	1.61	0.15
5 (cont)	Natural images	0	8			7	0.561	1.59	0.04
5 (cont)	Scrambled	0	8			7	0.539	1.53	0.17
6	Gray Screen	0	8		X	7	0.714	2.05	0.04

**Figure 3.**
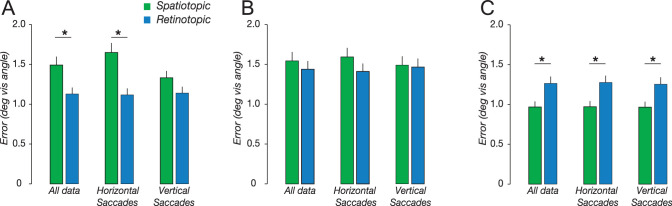
Memory accuracy in Experiment 1 (A), Experiment 2 (B), and Experiment 3 (C), with data broken down by saccade direction. *P < 0.05.

We analyzed horizontal and vertical saccades separately (Figure 3). We found that retinotopic memory was more accurate than spatiotopic memory for both horizontal [Cohen's d 1.18, t(7) = 4.66, *P* = 0.002] and vertical saccades [Cohen's d 1.55, t(7) = 2.76, *P* = 0.03]. A 2 × 2 repeated measures analysis of variance revealed a significant interaction between task (spatiotopic vs. retinotopic) and saccade direction (horizontal vs. vertical) [F(1,7) = 11.44, *P* = 0.011]. This interaction was due to an increase in accuracy of vertical relative to horizontal saccades in the spatiotopic task [Cohen's d 1.43 post hoc *t* test, t(7) = 2.73, *P* = 0.03]. The increased accuracy for vertical saccades could reflect the closer proximity of the edge of the screen to the remembered location on these trials (see Figure 1).

**Figure 4.**
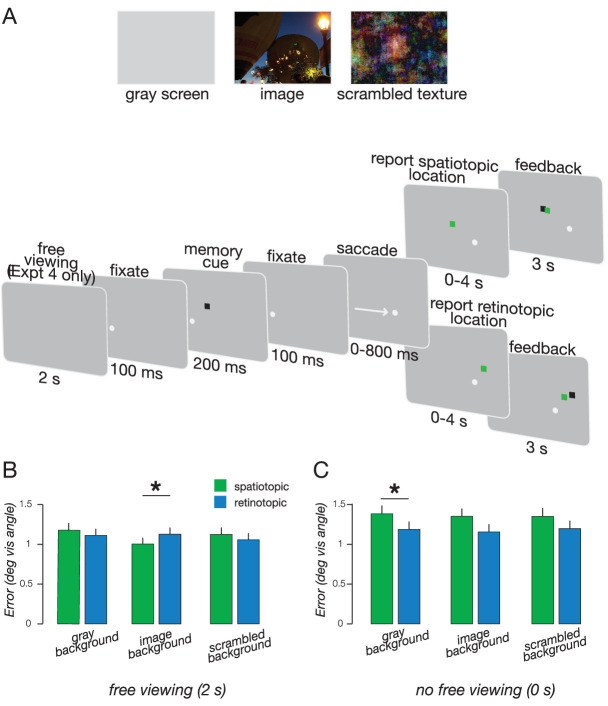
(A) Experiment design for Experiments 4 and 5. Three different background conditions (gray screen, image background, scrambled texture) were presented in a randomized order. There was only free viewing in Experiment 4. Example trial presented to the right. (B) Memory accuracy in Experiment 4 (free viewing). (C) Memory accuracy in Experiment 5 (no free viewing). *P < 0.05.

**Figure 5.**
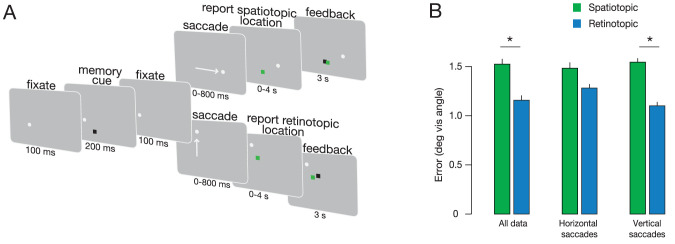
Experimental design and results for Experiment 6. (A), Example trials for Experiment 6 (B), Memory accuracy for Experiment 6 with data broken down by saccade direction. *P < 0.05.

In Experiment 1, we successfully replicated previous reports of a retinotopic memory advantage. However, retinotopic memory had a clear potential advantage in this experiment in the form of a retinal afterimage that might reflect the retinotopic location after the saccade. Experiment 2 tested whether the retinotopic superiority was partially a result of an after-image.

### Experiment 2

We tested whether the retinotopic advantage was in part due to a retinal afterimage. To decrease the retinal afterimage, a salient mask stimulus flashed on the screen after the memory cue. The presence of the mask dramatically reduced the magnitude of the retinotopic advantage (Figure 2). Repeated measures analysis of variance showed no main effect of task [F(1,7) = 0.75, *P* = 0.42], or saccade direction [F(1,7) = 0.21, *P* = 0.66], or an interaction [F(1,7) = 1.20, *P* = 0.31]. Similarly, we found no significant difference when we pooled across saccade direction [Cohen's d 0.288, t(7) = 0.86, *P* = 0.32] (Figure 3B). Additionally, there was no significant difference in memory precision owing to the task [Cohen's d 0.18, t(7) = 0.76, *P* = 0.47]. These results suggest that the retinotopic advantage observed in Experiment 1 and in previous reports (Golomb & Kanwisher, 2012b; Shafer-Skelton & Golomb, 2018) was in large part due to a retinal afterimage of the cue. It is worth noting that one previous study (Golomb & Kanwisher, 2012b) found that, although the mask eliminated the retinotopic advantage after a single saccade, after a second saccade, the retinotopic advantage returned. This raises the possibility that the retinotopic advantage is not driven exclusively by the retinal aftereffect, and additional components may be in play.

### Experiment 3

We tested whether the accuracy of spatial memory is influenced by visual context. Observers performed the same spatiotopic and retinotopic tasks as in Experiment 1, but the background consisted of large, static images, rather than a uniform gray (see Methods). Repeated measures analysis of variance identified a significant main effect of task [F(1,8) = 22.4, *P* = 0.0001] (Figure 2). A post hoc *t* test revealed that this was owing to higher accuracy in the spatiotopic task [Cohen's d −1.585, t(14) = −3.85, *P* = 0.002]. Notably, accuracy in the spatiotopic task improved by an average of 0.52°, resulting in a 35% increase in accuracy relative to Experiment 1. However, retinotopic accuracy was nearly identical to Experiment 1, decreasing by only 0.13° (10%). In the presence of background images spatiotopic memory improved for both horizontal [Cohen's d 1.55, t(8) = 3.85, *P* = 0.005] and vertical saccades [Cohen's d 1.12, t(8) = 2.93, = 0.02] (Figure 3C). Additionally, spatiotopic memory had significantly higher precision than retinotopic memory [t(8) = −2.8, *P* = 0.02]. These results suggest that the visual system naturally makes use of visual landmarks to improve the accuracy of spatiotopic representations across saccades.

### Experiments 4 and 5

The spatiotopic advantage observed in Experiment 3 raises a number of questions that we aimed to address in these experiments. First, is the spatiotopic advantage owing to the time observers were free to view the background image before the appearance of the memory cue? Second, is the spatiotopic advantage owing to the semantic content of the background image? To address these questions, we conducted experiments in which we tested the effects of background type and previewing time in subsequent memory. In Experiment 4, subjects freely viewed each background for 2 seconds before the appearance of the memory cue (Figure 4A). In contrast, Experiment 5 differed from Experiment 4 in that in Experiment 5 subjects were not given the opportunity to freely view the background, because the background image appeared at the same time as the memory cue.

When subjects viewed the background for 2 seconds before the cue appeared (Experiment 4) the task × background interaction was significant [F(2,14) = 5.96, *P* = 0.013] (Figure 4B). This was driven by a spatiotopic advantage for image background relative to no background condition [Cohen's d 0.91, t(7) = 2.46, *P* = 0.04], replicating the main finding in Experiment 3. This task × background interaction was not significant when subjects did not have time to view the background before the memory cue appeared (Experiment 5) [F(2,14) = 0.31, *P* = 0.74] (Figure 4C). Although there was no interaction, there was a significant effect of task (higher accuracy for retinotopic memory), but only for the gray background [Cohen's d 0.52, t(7) = 2.29, *P* = 0.04]. This result again replicated the main finding in Experiment 1 and demonstrates that the retinotopic advantage does not extend to situations in which semantic context is available to assist spatial memory. Comparing the two experiments, spatiotopic memory was more accurate with free viewing (Experiment 4) than without (Experiment 5) [Cohen's d 2.10, t(14) = 3.85, *P* = 0.002].

### Experiment 6

We wondered whether the retinotopic advantage was the result of a systematic spatial bias where responses were more accurate toward the edge of the screen. The screen edge may serve as a constant spatial landmark, used by subjects to localize the target. Specifically, in the retinotopic task the memory cues were located closer to the screen edge than in the spatiotopic task, which may introduce a systematic bias favoring retinotopic stimulus localization. To test this possibility, we ran a control experiment where the memory cue was presented toward the edge of the screen; the retinotopic response was brought toward the center of the screen, while the spatiotopic responses remained close to the edge (Figure 5A). We found the same pattern of results as in Experiment 1; there was higher accuracy for the retinotopic task than the spatiotopic task [Cohen's d 0.72, t(7) = 2.05, *P* = 0.04] (Figure 5B). This effect remained for the vertical saccades [Cohen's d 1.00, t(7) = 2.82, *P* = 0.03], but not the horizontal saccades [Cohen's d 0.40, t(7) = 1.14, *P* = 0.29]. We conclude that, although a bias away from the screen edge may be present in the original experiment, it is not the source of the retinotopic memory advantage.

## Discussion

In this series of experiments, we replicated the retinotopic memory advantage (Experiment 1). The retinotopic advantage disappeared when the retinal after-image was attenuated by a mask that appeared immediately after the cue (Experiment 2). When the visual context was changed to mimic those of real-life image viewing, spatiotopic memory became more accurate than retinotopic memory (Experiment 3 and Experiment 4), but only when subjects had sufficient time to process the visual context (Experiment 5). Taken together, our results indicate that, although a retinotopic reference frame may be the native reference frame for the visual system, under experimental conditions that mimic real-life scene viewing, the advantage is shifted in favor of a spatiotopic reference frame. We speculate that this change from retinotopic to spatiotopic advantage may reflect a shift from remapping to spatiotopic coding according to specific task demands.

### Visual context affects spatiotopic representations

The primate visual system is primarily retinotopic, yet visual perception is stable across frequent eye movements that shift the entire visual field. How is visual stability maintained across eye movements? One possibility is that during each eye movement visual representations are updated to compensate for retinal motion resulting directly from eye movements (Duhamel et al., 1992). Such updates may be implemented in various visual brain regions by receptive field remapping (Melcher & Colby, 2008). In remapping, receptive fields shift by the saccade vector around the time of the saccade, so that the perisaccadic receptive field encompasses the region in visual space that they will occupy after the saccade as well. Owing to remapping, the visual system can disambiguate displacement on the retina caused by the change in the visual scene from that caused by an eye movement (Sommer & Wurtz, 2006). Because remapping is likely an imperfect coordinate transformation, spatial memories should be degraded and less accurate after each saccade.

The primate brain is thought to represent stimulus location in multiple reference frames that are not directly linked to position on the retina, including head-centered, world-centered, and object-centered coordinates (for a review, see Colby, 1998). It is possible that the brain uses one or more of these nonretinal reference frames to accomplish perceptual stability during eye movements. Our experiment cannot distinguish between these different references frames, because we did not vary head or body position. Hence, we use the general term “spatiotopic” to refer to a coordinate system that is independent of eye position (Crespi et al., 2011; d'Avossa et al., 2007; McKyton & Zohary, 2007). A spatiotopic representation can be computed from multiple sources of information, including proprioception (Allin, Velay, & Bouquerel, 1996; Balslev & Miall, 2008), eye position gain fields (Andersen & Mountcastle, 1983; Merriam, Gardner, Movshon, & Heeger, 2013; Siegel, Raffi, Phinney, Turner, & Jandó, 2003), and visual contextual cues (McConkie & Currie, 1996). Because a spatiotopic reference frame does not undergo abrupt updating, the accuracy of spatiotopic coding, unlike remapping, should be independent of the number of eye movements that occur in a trial.

These two mechanisms for visual stability—remapping and spatiotopic coding—are not mutually exclusive, and the visual system may depend on both. Moreover, it is conceivable that the relative prominence of each mechanism may depend on task demands. Indeed, the accuracy of spatial memory decreases with increasing numbers of saccades (Bock, Goltz, Bélanger, & Steinbach, 1995; Collins, 2010; Golomb & Kanwisher, 2012b), an effect that has been modeled as a weighted combination of spatiotopic coding and receptive field remapping (Poletti, Burr, & Rucci, 2013). These results suggest that both remapping and spatiotopic coding are computed on each trial, and both contribute to perception, but to varying degrees. Accordingly, the relative contribution of each mechanism may depend on the number of intervening saccades (Sun & Goldberg, 2016). After a single saccade, receptive field remapping may be the primary mechanism underlying visual stability, whereas spatiotopic coding may become prominent after a sequence of multiple saccades. In our study, the memory task involved a single intervening saccade; therefore, spatial memory should rely entirely on remapping, rather than spatiotopic coding. However, the weighting assigned to each mechanism may depend on other parameters aside from the number of intervening saccades. For example, electrophysiological studies have shown that, when the visual scene consists of many visual stimuli, remapping does not take place (Churan, Guitton, & Pack, 2011; Marino & Mazer, 2018; Zanos, Mineault, Nasiotis, Guitton, & Pack, 2015). Moreover, visual landmarks improve target localization, suggesting that the visual system can rely on retinal cues to keep track of target locations across eye movements (Zhang & Golomb, 2018). These observations are consistent with the notion that remapping many stimulus locations simultaneously would place too great a burden on system resources (Cavanagh, Hunt, Afraz, & Rolfs, 2010; Neupane, Guitton, & Pack, 2020). According to this reasoning, when multiple stimuli are visible, remapping is not computed at all; as a result, its weighting would be at or near zero. It is likely that our experiments created a similar scenario. When a naturalistic scene is present, the remapping mechanism may become overwhelmed by the rich visual stimulation. Instead of remapping, the visual system may resort to spatiotopic coding, relying instead on ocular proprioception and/or visual landmarks. This strategy can take advantage of the multitude of stimuli enabling a more accurate triangulation of the cue position. The degree to which the two mechanisms contribute to perceptual stability remains to be determined.

### Is retinotopic memory more accurate than spatiotopic memory?

Because all visual input is originally obtained in retinal coordinates, spatiotopic information must result from a coordinate transformation on the input. It is, therefore, perhaps unsurprising that retinotopic representations would be more accurate than spatiotopic representations (Golomb & Kanwisher, 2012b). However, Experiment 2 shows that the retinotopic representation per se is actually not more accurate than the spatiotopic representation. In the absence of a mask, retinotopic memory has an obvious advantage: the retinal afterimage lingers long after the cue has disappeared. It may be argued that in real life conditions, stimuli are usually not masked, and that in such situations retinotopic memory is more accurate. In Experiments 3 and 4, we found that, on the contrary, in a richer, more natural visual context spatiotopic memory takes advantage of visual landmarks and outperforms retinotopic memory.

### Advantage of spatiotopic memory in natural context

We found that when the memory cue is presented in the context of natural scenes, spatiotopic memory is superior to retinotopic memory. This is true when all aspects of the experimental design were kept constant, except for the addition of information-rich background images on the screen. If the spatiotopic representation is a transformation of retinotopic information, how could it be more accurate than the retinotopic representation? We propose that spatiotopic coding makes use of additional information that retinotopic coding ignores, resulting in a more accurate initial encoding of the cue location. This is supported by comparing results of Experiment 1 and Experiment 3 through 5. Retinotopic memory accuracy across all these experiments is relatively stable, while spatiotopic memory is elevated in Experiment 3 through 5 (as evident by the decrease in error). This finding suggests, first, that the natural images did not disrupt the retinal afterimage, unlike the mask used in Experiment 2. Second, this pattern of results suggests that spatiotopic coding takes advantage of the visual context to localize the memory cue, although retinotopic coding is unaffected by the additional information. How does context assist spatiotopic memory? We have previously shown that similarity between population response patterns in retinotopic visual cortex reflects proximity between stimuli at different locations (Roth, 2016). Storing those proximity estimates between the cue and nearby landmarks would therefore suffice for the system to triangulate the spatiotopic location of the cue, as long as the background is present. One important question relates to whether there is a critical temporal window for integration of the visual context in relation to the saccade. In our experiment, we only explored two temporal windows (in Experiments 3 and 4 subjects could view the background for 2 seconds, whereas in Experiment 5 subjects had no time for free viewing). Although we found that the difference between these two viewing conditions had an impact on memory fidelity, we did not fully explore the temporal dynamics of visual integration in this task. Future studies should test temporal integration with finer granularity.

### Potential spatial biases

Comparing memory accuracy across reference frames is complicated somewhat by the existence of systematic spatial biases that can exist in both spatial localization and the motoric aspects of response generation. For example, it has been shown that subjects exhibit a systematic bias toward the initial mouse pointer position (Prime, Niemeier, & Crawford, 2006). We note that such a bias, if present in our data, would not affect the comparison of spatiotopic and retinotopic memory, because the initial mouse position was equidistant from spatiotopic and retinotopic cue positions. We were concerned, however, about systematic biases related to the edge of the screen. For example, in Experiment 1 the correct retinotopic target locations are always near the edge of the screen, which could facilitate either target localization or pointing behavior if a systematic bias exists. However, Experiment 6 demonstrated that the retinotopic advantage is not due to such a bias.

### Effect of viewing conditions across multiple saccades

In this study, we identified factors that affect location memory after a single saccade. However, the same factors may additionally influence the rate at which spatiotopic memory degrades across multiple saccades. For example, as discussed elsewhere in this article, natural scene backgrounds may provide enough context to prevent remapping from occurring, instead causing the visual system to rely on spatiotopic coding, in which case accuracy should not depend on the number of intervening saccades. Further studies are needed to investigate how masking and visual context affect retinotopic and spatiotopic memory across multiple saccades.
